# Supplementary material for: Mechanistic computational modeling of sFLT1 secretion dynamics
Source: PLoS Comput Biol. 2025 Aug 18;21(8):e1013324. doi: 10.1371/journal.pcbi.1013324 (PMC12370208; doi:10.1371/journal.pcbi.1013324)
Supplement: S1 Text — (PDF) [file pcbi.1013324.s002.pdf]

S1 Text. Supplemental Methods

Here we derive additional equations and relationships used in analysis of these ODE and DDE models of sFLT1 secretion. Some equations are repeated from the main text for clarity, and are numbered as in the main text.

Ordinary differential equations (ODEs). The rate of change of intracellular sFLT1 ($\frac{dI(t)}{dt}$) is the balance of production, secretion, and intracellular degradation:

$$\frac{dI(t)}{dt} = \alpha - \beta \cdot I(t) - \gamma \cdot I(t) \qquad \text{(Eq. 1)}$$

The rate of change of extracellular sFLT1 ($\frac{dX(t)}{dt}$) is the balance of secretion and extracellular degradation:

$$\frac{dX(t)}{dt} = \beta \cdot I(t) - \delta \cdot X(t) \qquad \text{(Eq. 2)}$$

Units of *I*, *X*: #/cell (number of proteins per cell)

Units of α: #/cell/h (number of proteins per cell per hour)

Units of β, γ, δ: 1/h (inverse hours)

Delay differential equations (DDEs). These equations apply a time delay, τ, to intracellular sFLT1 when calculating secretion rate and intracellular degradation rate:

$$\frac{dI(t)}{dt} = \alpha - \beta \cdot I(t-\tau) - \gamma \cdot I(t-\tau) \qquad \text{(Eq. 3)}$$

$$\frac{dX(t)}{dt} = \beta \cdot I(t-\tau) - \delta \cdot X(t) \qquad \text{(Eq. 4)}$$

Units of τ: h (hours)

Observed constraints. Optimization of our model to experimental data yielded the following constants:

$$c_1 = \alpha\beta$$

i.e., the product of production rate and secretion rate constant is constrained to a single value.

$$c_2 = \beta + \gamma$$

i.e., the sum of secretion and intracellular degradation rate constants is constrained to a single value.

Theoretical steady state (I_{SS}, X_{SS}) of the ODE and DDE systems. At steady state:

$\frac{dI(t)}{dt} = \frac{dX(t)}{dt} = 0$, $I(t) = I_{SS}$, and $X(t) = X_{SS}$.

From Eq. 1:

$$0 = \alpha - (\beta+\gamma) \cdot I_{SS} \Rightarrow \alpha = (\beta+\gamma) \cdot I_{SS} \Rightarrow I_{SS} = \frac{\alpha}{\beta+\gamma} \qquad \text{(Eq. 6)}$$

From Eq. 2:

$$0 = \beta \cdot I_{SS} - \delta \cdot X_{SS} \Rightarrow \beta \cdot I_{SS} = \delta \cdot X_{SS} \Rightarrow X_{SS} = \frac{\alpha\beta}{\delta(\beta+\gamma)} \qquad \text{(Eq. 7)}$$

In terms of the observed constraints $c_1 = \alpha\beta$ and $c_2 = \beta + \gamma$:

$$I_{SS} = \frac{\alpha}{\beta+\gamma} = \frac{\alpha}{c_2} = \frac{c_1}{\beta c_2} \qquad \text{(Eq. 6a)}$$

$$X_{SS} = \frac{\alpha\beta}{\delta(\beta+\gamma)} = \frac{c_1}{\delta c_2} = I_{SS}\frac{\beta}{\delta} \qquad \text{(Eq. 7a)}$$

For the DDE system, $I(t-\tau) = I(t) = I_{SS}$, and $X(t-\tau) = X(t) = X_{SS}$. Thus, we get the same results as Eq. 6a and Eq. 7a.

Characteristic time to half-steady-state for intracellular sFLT1 (T_{50_I}) for the ODE system. Assuming first order secretion rate constant β and first order intracellular degradation rate constant γ, *I* has a combined first order elimination rate constant $\beta + \gamma$. From first principles of kinetics, accumulation of *I* to I_{SS} is:

$$I(t) = I_{SS} \cdot (1 - e^{-(\beta+\gamma)\cdot t})$$

At $t = T_{50\_I}$, when $I(T_{50\_I}) = \frac{I_{SS}}{2}$:

$$\frac{I_{SS}}{2} = I_{SS} \cdot (1 - e^{-(\beta+\gamma)\cdot T_{50\_I}}) \Rightarrow \frac{1}{2} = 1 - e^{-(\beta+\gamma)\cdot T_{50\_I}} \Rightarrow e^{-(\beta+\gamma)\cdot T_{50\_I}} = \frac{1}{2} \Rightarrow$$

$$-(\beta+\gamma) \cdot T_{50\_I} = ln\,\frac{1}{2} \Rightarrow$$

$$T_{50\_I} = \frac{ln\,2}{\beta+\gamma} \qquad \text{(Eq. 8)}$$

Characteristic time to half-steady-state for extracellular sFLT1 (T_{50_X}) for the ODE system. From first principles of kinetics, assuming first order extracellular degradation rate constant δ accumulation of *X* to X_{SS} is:

$$X(t) = X_{SS} \cdot (1 - e^{-\delta\cdot t})$$

At $t = T_{50\_X}$, when $I(T_{50\_X}) = \frac{X_{SS}}{2}$:

$$\frac{X_{SS}}{2} = X_{SS} \cdot (1 - e^{-\delta\cdot T_{50\_X}}) \Rightarrow \frac{1}{2} = 1 - e^{-\delta\cdot T_{50\_X}} \Rightarrow e^{-\delta T_{50\_X}} = \frac{1}{2} \Rightarrow$$

$$-\delta \cdot T_{50\_I} = ln\,\frac{1}{2} \Rightarrow$$

$$T_{50\_X} = \frac{ln\,2}{\delta} \qquad \text{(Eq. 9)}$$

Process fluxes. These non-negative fluxes represent the rates over time of each modeled process in #/cell/h, and are the constituent rate terms from the right hand side of the DDE equations given earlier. The fluxes for the ODEs are identical for $\tau = 0$. Prod = production, Secr = secretion, IDeg = intracellular degradation, XDeg = extracellular degradation.

$$\Phi_{Prod}(t) = \alpha$$
$$\Phi_{Secr}(t) = \beta \cdot I(t-\tau)$$
$$\Phi_{IDeg}(t) = \gamma \cdot I(t-\tau)$$
$$\Phi_{XDeg}(t) = \delta \cdot X(t)$$

Steady state secretion flux (Φ_{Secr}). At steady state, from Eq. 6a, $I(t-\tau) = I_{SS} = \frac{\alpha}{\beta+\gamma}$.

Therefore:

$$\Phi_{Secr} = \beta \cdot I_{SS} = \frac{\alpha\beta}{\beta+\gamma} = \frac{c_1}{c_2}$$

Steady state relationship between intracellular degradation flux (Φ_{IDeg})and production flux (Φ_{Prod}). At steady state, from Eq. 6a, $I(t-\tau) = I_{SS} = \frac{\alpha}{c_2} = \frac{\Phi_{Prod}}{c_2}$. Therefore:

$$\Phi_{IDeg} = \gamma \cdot \frac{\Phi_{Prod}}{c_2} = \frac{c_2-\beta}{c_2} \cdot \Phi_{Prod} = (1-\frac{\beta}{c_2}) \cdot \Phi_{Prod} = \Phi_{Prod} - \frac{\alpha\beta}{c_2} \Rightarrow$$
$$\Phi_{IDeg} = \Phi_{Prod} - \frac{c_1}{c_2}$$

Asymptotic behavior of steady state intracellular degradation flux (Φ_{IDeg}). Since Φ_{IDeg} is non-negative, $min(\Phi_{IDeg}) = 0$, and $\Phi_{Prod} \geq \frac{c_1}{c_2}$.

When $\Phi_{Prod} \gg \frac{c_1}{c_2}$, $\Phi_{IDeg} = \Phi_{Prod} - \frac{c_1}{c_2} \rightarrow \Phi_{Prod}$.

i.e. maximum intracellular degradation is equal to production, and this occurs when the secretion rate constant is zero ($\beta \rightarrow 0$).

Theoretical lower bound of production flux (Φ_{Prod}). Φ_{Prod} increases monotonically with Φ_{IDeg}, so $min(\Phi_{Prod})$ occurs when $\Phi_{IDeg} = min(\Phi_{IDeg})$. Therefore:

$$min(\Phi_{Prod}) = min(\Phi_{IDeg}) + \frac{c_1}{c_2} = 0 + \frac{c_1}{c_2} = \frac{c_1}{c_2}$$

i.e. production is minimized when there is no intracellular degradation, only secretion.

Steady state relationship between intracellular sFLT1 (I_{SS}) and intracellular degradation flux (Φ_{IDeg}).

$$I_{SS} = \frac{\Phi_{Prod}}{c_2} \Rightarrow \Phi_{Prod} = c_2 I_{SS}$$

$$\Phi_{IDeg} = \Phi_{Prod} - \frac{c_1}{c_2} = c_2 I_{SS} - \frac{c_1}{c_2} \Rightarrow c_2 I_{SS} = \Phi_{IDeg} + \frac{c_1}{c_2} \Rightarrow$$

$$I_{SS} = \frac{\Phi_{IDeg}}{c_2} + \frac{c_1}{{c_2}^2}$$

Asymptotic behavior of steady state intracellular sFLT1 (I_{SS}). Since both I_{SS} and Φ_{Prod} are non-negative and directly proportional, $min(I_{SS})$ occurs when $\Phi_{Prod} = min(\Phi_{Prod})$. Therefore:

$$min(I_{SS}) = min(\Phi_{Prod})/c_2 = \frac{c_1/c_2}{c_2} \Rightarrow min(I_{SS}) = \frac{c_1}{{c_2}^2}$$

This is also consistent with $I_{SS} = \frac{\Phi_{IDeg}}{c_2} + \frac{c_1}{{c_2}^2}$ as $\Phi_{IDeg} \to 0$.

When $\Phi_{IDeg} \gg \frac{c_1}{c_2}$, $\Phi_{IDeg} + \frac{c_1}{c_2} \to \Phi_{IDeg}$ and $I_{SS} = \frac{1}{c_2}(\Phi_{IDeg} + \frac{c_1}{c_2}) \to \frac{\Phi_{IDeg}}{c_2}$.
