# Supplementary material for: Mechanistic computational modeling of sFLT1 secretion dynamics
Source: PLoS Comput Biol. 2025 Aug 18;21(8):e1013324. doi: 10.1371/journal.pcbi.1013324 (PMC12370208; doi:10.1371/journal.pcbi.1013324)
Supplement: S2 Text — (PDF) [file pcbi.1013324.s003.pdf]

S2 Text. Supplemental Results

General analysis of ODE model of secretion. Analysis of model solution dynamics (**S1A Fig**) shows that in response to a step increase in the rate of production from 0 to α at time $t = 0$, intracellular sFLT1 increases hyperbolically to steady state, and extracellular sFLT1 increases to its steady state with a delay relative to pure first-order kinetics (which would result in X reaching $X_{SS}/2$ at a normalized time of 1); this delay is due to the time needed for production and secretion to occur. The system has an equal but opposite response to a step decrease in production. Phase plane analysis confirms that intracellular, extracellular, and total sFLT1 increase monotonically when production increases and decrease monotonically when production decreases, and that intracellular sFLT1 approaches its steady state faster than extracellular sFLT1 (**S1B Fig**).

General analysis of DDE model of secretion. In an example solution to the base DDE model (**S2A-B Fig)**, intracellular sFLT1 initially responds to a step increase in production by overshooting its theoretical steady state value I_{SS}, while extracellular sFLT1 remains at 0 until $t = \tau$. As sFLT1 secretion begins, extracellular sFLT1 accumulates, while the intracellular sFLT1 first slows in accumulation rate and then decreases sharply below I_{SS}. Because of the delay, sFLT1 secretion initially continues to increase even as intracellular sFLT1 decreases, and extracellular sFLT1 also exceeds its theoretical steady state value X_{SS}. As intracellular sFLT1 falls, its production exceeds its clearance, and intracellular sFLT1 once again begins to accumulate and shows damped oscillations converging towards I_{SS}. These oscillations drive similar damped oscillations in extracellular sFLT1 converging towards X_{SS}, and because the

oscillations are out of phase, total sFLT1 also oscillates rather than monotonically approaching steady state.

Phase plane analysis of DDE model for varying secretion delay. While the ODE model generated similar solution dynamics and phase plane portraits across a large region of parameter space, the DDE model solution shape varied significantly with the delay τ, which changed both the amplitude and frequency of oscillations (**S2C-D Fig**). Some parameter regimes created dynamics that could not converge to steady state because they generated situations where intracellular sFLT1 clearance flux exceeded the available amount of intracellular sFLT1, a situation incompatible with conservation of mass. We therefore concluded that some parameter combinations were not physically permissible using the DDE model, and we excluded those combinations from future analysis.

Sensitivity to values of constraints c_1 and c_2. To better characterize the impact of observed constraints (c_1, c_2), we varied one of these values while holding the other constant and simulated constitutive secretion. First, we allowed α and β to vary while maintaining constant $c_1 = \alpha\beta = 7270$ #/cell/h^2, resulting in a range of values for $c_2 = \beta + \gamma$. When c_1 is fixed, larger values of c_2 generate lower intracellular and extracellular sFLT1 levels (**S10A Fig**). Under these conditions, increasing α causes a linear increase in intracellular sFLT1 but a hyperbolic increase in extracellular sFLT1, while both intracellular and extracellular sFLT1 are inversely proportional to β and c_2 (**S10B Fig**).

Then, we allowed (β, γ) to vary while maintaining constant $c_2 = \beta + \gamma = 0.173$ h^{-1}, resulting in a range of values for $c_1 = \alpha\beta$. When c_2 is fixed, intracellular sFLT1 remains constant independent of c_1, but extracellular sFLT1 increases as c_1 increases (**S10C Fig**). The extracellular sFLT1 concentration increases linearly with increasing c_1 and β, and decreases linearly with increasing γ (**S10D Fig**).
