# Supplementary material for: Mechanistic computational modeling of sFLT1 secretion dynamics
Source: PLoS Comput Biol. 2025 Aug 18;21(8):e1013324. doi: 10.1371/journal.pcbi.1013324 (PMC12370208; doi:10.1371/journal.pcbi.1013324)
Supplement: S3 Text — (PDF) [file pcbi.1013324.s004.pdf]

S3 Text. Supplemental References

MATLAB software. MATLAB and Optimization Toolbox Release 2022a, The MathWorks, Inc., Natick, Massachusetts, United States.

R software. References were generated in R using report::cite_packages.

Bengtsson H (2022). R.matlab: Read and Write MAT Files and Call MATLAB from Within R. R package version 3.7.0, https://CRAN.R-project.org/package=R.matlab.

Garnier, Simon, Ross, Noam, Rudis, Robert, Camargo, Pedro A, Sciaini, Marco, Scherer, Cédric (2023). viridis(Lite) - Colorblind-Friendly Color Maps for R. doi:10.5281/zenodo.4678327 https://doi.org/10.5281/zenodo.4678327, viridisLite package version 0.4.2, https://sjmgarnier.github.io/viridis/.

Garnier, Simon, Ross, Noam, Rudis, Robert, Camargo, Pedro A, Sciaini, Marco, Scherer, Cédric (2024). viridis(Lite) - Colorblind-Friendly Color Maps for R. doi:10.5281/zenodo.4679423 https://doi.org/10.5281/zenodo.4679423, viridis package version 0.6.5, https://sjmgarnier.github.io/viridis/.

Grolemund G, Wickham H (2011). "Dates and Times Made Easy with lubridate." Journal of Statistical Software, 40(3), 1-25. https://www.jstatsoft.org/v40/i03/.

Gu Z, Eils R, Schlesner M (2016). "Complex heatmaps reveal patterns and correlations in multidimensional genomic data." Bioinformatics. doi:10.1093/bioinformatics/btw313 https://doi.org/10.1093/bioinformatics/btw313. Gu Z (2022). "Complex Heatmap Visualization." iMeta. doi:10.1002/imt2.43 https://doi.org/10.1002/imt2.43.

Gu Z, Gu L, Eils R, Schlesner M, Brors B (2014). "circlize implements and enhances circular visualization in R." Bioinformatics, 30, 2811-2812.

Meschiari S (2022). latex2exp: Use LaTeX Expressions in Plots. R package version 0.9.6, https://CRAN.R-project.org/package=latex2exp.

Müller K, Wickham H (2023). tibble: Simple Data Frames. R package version 3.2.1, https://CRAN.R-project.org/package=tibble.

Pedersen T (2024). patchwork: The Composer of Plots. R package version 1.2.0, https://CRAN.R-project.org/package=patchwork.

R Core Team (2024). R: A Language and Environment for Statistical Computing. R Foundation for Statistical Computing, Vienna, Austria. https://www.R-project.org/.

Schloerke B, Cook D, Larmarange J, Briatte F, Marbach M, Thoen E, Elberg A, Crowley J (2024). GGally: Extension to 'ggplot2'. R package version 2.2.1, https://CRAN.R-project.org/package=GGally.

Slowikowski K (2024). ggrepel: Automatically Position Non-Overlapping Text Labels with 'ggplot2'. R package version 0.9.6, https://CRAN.R-project.org/package=ggrepel.

Wickham H (2011). "testthat: Get Started with Testing." The R Journal, 3, 5-10. https://journal.r-project.org/archive/2011-1/RJournal_2011-1_Wickham.pdf.

Wickham H (2016). ggplot2: Elegant Graphics for Data Analysis. Springer-Verlag New York. ISBN 978-3-319-24277-4, https://ggplot2.tidyverse.org.

Wickham H (2023). forcats: Tools for Working with Categorical Variables (Factors). R package version 1.0.0, https://CRAN.R-project.org/package=forcats.

Wickham H (2023). stringr: Simple, Consistent Wrappers for Common String Operations. R package version 1.5.1, https://CRAN.R-project.org/package=stringr.

Wickham H, Averick M, Bryan J, Chang W, McGowan LD, François R, Grolemund G, Hayes A, Henry L, Hester J, Kuhn M, Pedersen TL, Miller E, Bache SM, Müller K, Ooms J,

Robinson D, Seidel DP, Spinu V, Takahashi K, Vaughan D, Wilke C, Woo K, Yutani H (2019). “Welcome to the tidyverse.” Journal of Open Source Software, 4(43), 1686. doi:10.21105/joss.01686 https://doi.org/10.21105/joss.01686.

Wickham H, François R, Henry L, Müller K, Vaughan D (2023). dplyr: A Grammar of Data Manipulation. R package version 1.1.4, https://CRAN.R-project.org/package=dplyr.

Wickham H, Henry L (2023). purrr: Functional Programming Tools. R package version 1.0.2, https://CRAN.R-project.org/package=purrr.

Wickham H, Hester J, Bryan J (2024). readr: Read Rectangular Text Data. R package version 2.1.5, https://CRAN.R-project.org/package=readr.

Wickham H, Pedersen T, Seidel D (2023). scales: Scale Functions for Visualization. R package version 1.3.0, https://CRAN.R-project.org/package=scales.

Wickham H, Vaughan D, Girlich M (2024). tidyr: Tidy Messy Data. R package version 1.3.1, https://CRAN.R-project.org/package=tidyr.

Wilke C, Wiernik B (2022). gridtext: Improved Text Rendering Support for 'Grid' Graphics. R package version 0.1.5, https://CRAN.R-project.org/package=gridtext.
