# Supplementary material for: Mechanistic computational modeling of sFLT1 secretion dynamics
Source: PLoS Comput Biol. 2025 Aug 18;21(8):e1013324. doi: 10.1371/journal.pcbi.1013324 (PMC12370208; doi:10.1371/journal.pcbi.1013324)
Supplement: S1 Table — Note that model M1 is equivalent to the “ODE-based model” and model M2 is equivalent to the “DDE-based model” described above. (PDF) [file pcbi.1013324.s005.pdf]

S1 Table. Complete set of equations for candidate models. Note that model M1 is equivalent to the "ODE-based model" and model M2 is equivalent to the "DDE-based model" described above.

Model	ε	κ	τ	Equations
M1	N	N	N	$\frac{dI(t)}{dt} = \alpha - \beta \cdot I(t) - \gamma \cdot I(t)$<br>$\frac{dX(t)}{dt} = \beta \cdot I(t) - \delta \cdot X(t)$
M2	N	N	Y	$\frac{dI(t)}{dt} = \alpha - \beta \cdot I(t-\tau) - \gamma \cdot I(t-\tau)$<br>$\frac{dX(t)}{dt} = \beta \cdot I(t-\tau) - \delta \cdot X(t)$
M3	N	Y	N	$\frac{dI(t)}{dt} = \alpha e^{-\kappa t} - \beta \cdot I(t) - \gamma \cdot I(t)$<br>$\frac{dX(t)}{dt} = \beta \cdot I(t) - \delta \cdot X(t)$
M4	N	Y	Y	$\frac{dI(t)}{dt} = \alpha e^{-\kappa t} - \beta \cdot I(t-\tau) - \gamma \cdot I(t)$<br>$\frac{dX(t)}{dt} = \beta \cdot I(t-\tau) - \delta \cdot X(t)$
M5	Y	N	N	$\frac{dI(t)}{dt} = \alpha - \beta \cdot I(t) - \gamma \cdot I(t) + \epsilon \cdot X(t)$<br>$\frac{dX(t)}{dt} = \beta \cdot I(t) - \delta \cdot X(t) - \epsilon \cdot X(t)$
M6	Y	N	Y	$\frac{dI(t)}{dt} = \alpha - \beta \cdot I(t-\tau) - \gamma \cdot I(t-\tau) + \epsilon \cdot X(t)$<br>$\frac{dX(t)}{dt} = \beta \cdot I(t-\tau) - \delta \cdot X(t) - \epsilon \cdot X(t)$
M7	Y	Y	N	$\frac{dI(t)}{dt} = \alpha e^{-\kappa t} - \beta \cdot I(t) - \gamma \cdot I(t) + \epsilon \cdot X(t)$

				$\frac{dX(t)}{dt} = \beta \cdot I(t) - \delta \cdot X(t) - \epsilon \cdot X(t)$
M8	Y	Y	Y	$\frac{dI(t)}{dt} = \alpha e^{-\kappa t} - \beta \cdot I(t-\tau) - \gamma \cdot I(t) + \epsilon \cdot X(t)$ $\frac{dX(t)}{dt} = \beta \cdot I(t-\tau) - \delta \cdot X(t) - \epsilon \cdot X(t)$
