# Supplementary material for: Mechanistic computational modeling of sFLT1 secretion dynamics
Source: PLoS Comput Biol. 2025 Aug 18;21(8):e1013324. doi: 10.1371/journal.pcbi.1013324 (PMC12370208; doi:10.1371/journal.pcbi.1013324)
Supplement: S2 Table — Secretion scenario: C = constitutive, P = pulse-chase; Assay: E = ELISA, W = Western blot/ autoradiography; Time points: * = flattened by normalization, † = omitted (see Methods for rationale). (PDF) [file pcbi.1013324.s006.pdf]

Supplemental Table S2. Characteristics of sFLT1 datasets used for mechanistic model optimization. Secretion scenario: C = constitutive, P = pulse-chase; Assay: E = ELISA, W = Western blot / autoradiography; Time points: * = flattened by normalization, † = omitted (see Methods for rationale).

Dataset	Reference	Secretion scenario	Assay	X unit	X time points (h)	I unit	I time points (h)	# of data points (AIC_C)
Hornig	1	C	E	ng/mL	0*, 3†, 6, 9, 12, 24, 48, 72	NA	NA	6
Jung	2	P	W	$\frac{X}{X_{8h}}$	0*, 2, 4, 6, 8*, 10†	$\frac{I}{I_{0h}}$	0*, 2, 4, 6, 8, 10	8
Kinghorn	3	C	W	$\frac{X}{X_{24h}}$	0, 1, 2, 4, 8, 12, 24*	$\frac{I}{I_{0h}}$	0*, 1, 2, 4, 8, 12, 24	12

References

1. Hornig, C. et al. Release and Complex Formation of Soluble VEGFR-1 from Endothelial Cells and Biological Fluids. Lab Invest 80, 443–454 (2000).

2. Jung, J.-J. et al. Secretion of Soluble Vascular Endothelial Growth Factor Receptor 1 (sVEGFR1/sFlt1) Requires Arf1, Arf6, and Rab11 GTPases. PLoS One 7, e44572 (2012).

3. Kinghorn, K. et al. A defined clathrin-mediated trafficking pathway regulates sFLT1/VEGFR1 secretion from endothelial cells. Angiogenesis 27, 67–89 (2024).
