# Supplementary material for: Mechanistic computational modeling of sFLT1 secretion dynamics
Source: PLoS Comput Biol. 2025 Aug 18;21(8):e1013324. doi: 10.1371/journal.pcbi.1013324 (PMC12370208; doi:10.1371/journal.pcbi.1013324)
Supplement: S3 Table — (PDF) [file pcbi.1013324.s007.pdf]

S3 Table. Sampling distributions for initial parameter values and bounds for final parameter values during mechanistic model optimization.

Parameter	Description	Distribution	Sampling bounds		Optimization bounds		Units
			Lower	Upper	Lower	Upper	
α	Production rate constant	Log uniform	5×10^{3}	5×10^{6}	ODE: 1×10^{3}	ODE: 1×10^{7}	#/cell/h
					other: 1×10^{-4}	other: 1×10^{7}	
β	Secretion rate constant	Log uniform	1×10^{-3}	1	ODE: 1×10^{-6}	ODE: 1	1/h
					other: 1×10^{-4}	other: 1×10^{7}	
γ	Intracellular degradation rate constant	Log uniform	1×10^{-3}	1	ODE: 1×10^{-6}	ODE: 1	1/h
					other: 1×10^{-4}	other: 1×10^{7}	
δ	Extracellular degradation rate constant	Log uniform	1×10^{-3}	1	ODE: 1×10^{-6}	ODE: 1	1/h
					other: 1×10^{-4}	other: 1×10^{7}	
ϵ	Internalization rate constant	Log uniform	1×10^{-3}	1	1×10^{-4}	1×10^{7}	1/h
κ	Production decay [chase] constant	Uniform	0	10	1×10^{-4}	1×10^{7}	1/h
τ	Maturation time delay	Uniform	1	4	1×10^{-4}	1×10^{7}	h
