# Supplementary material for: Mechanistic computational modeling of sFLT1 secretion dynamics
Source: PLoS Comput Biol. 2025 Aug 18;21(8):e1013324. doi: 10.1371/journal.pcbi.1013324 (PMC12370208; doi:10.1371/journal.pcbi.1013324)
Supplement: S2 Fig — (A) Solution of the DDE model as a function of time showing intracellular (I) and extracellular (X) sFLT1 protein dynamics in response to step changes in sFLT1 production (α). Time is normalized to T50_X, the half-life of X (Eq. 4). I,X are normalized to their steady state values ISS, XSS (Eq. 1–2). (B) Phase portrait of the shown DDE model solution. Points are evenly spaced every two time units and colored by time as in panel A. Arrowheads indicate the direction of increasing time starting with t = 0 in the lower left. The dashed identity line represents potential steady states (I=βδ·X); total sFLT1 increases in regions above this line and decreases below. Parameter values: α=10,000, β=0.1, γ=0.1, δ=0.1, τ=T50_X. (C) Example DDE solutions as a function of time and (D) as a phase portrait for maturation delays τ=(0, 0.5, 1, 1.5, 2)·T50_X. For discussion of this figure, see S2 Text. (PDF) [file pcbi.1013324.s009.pdf]

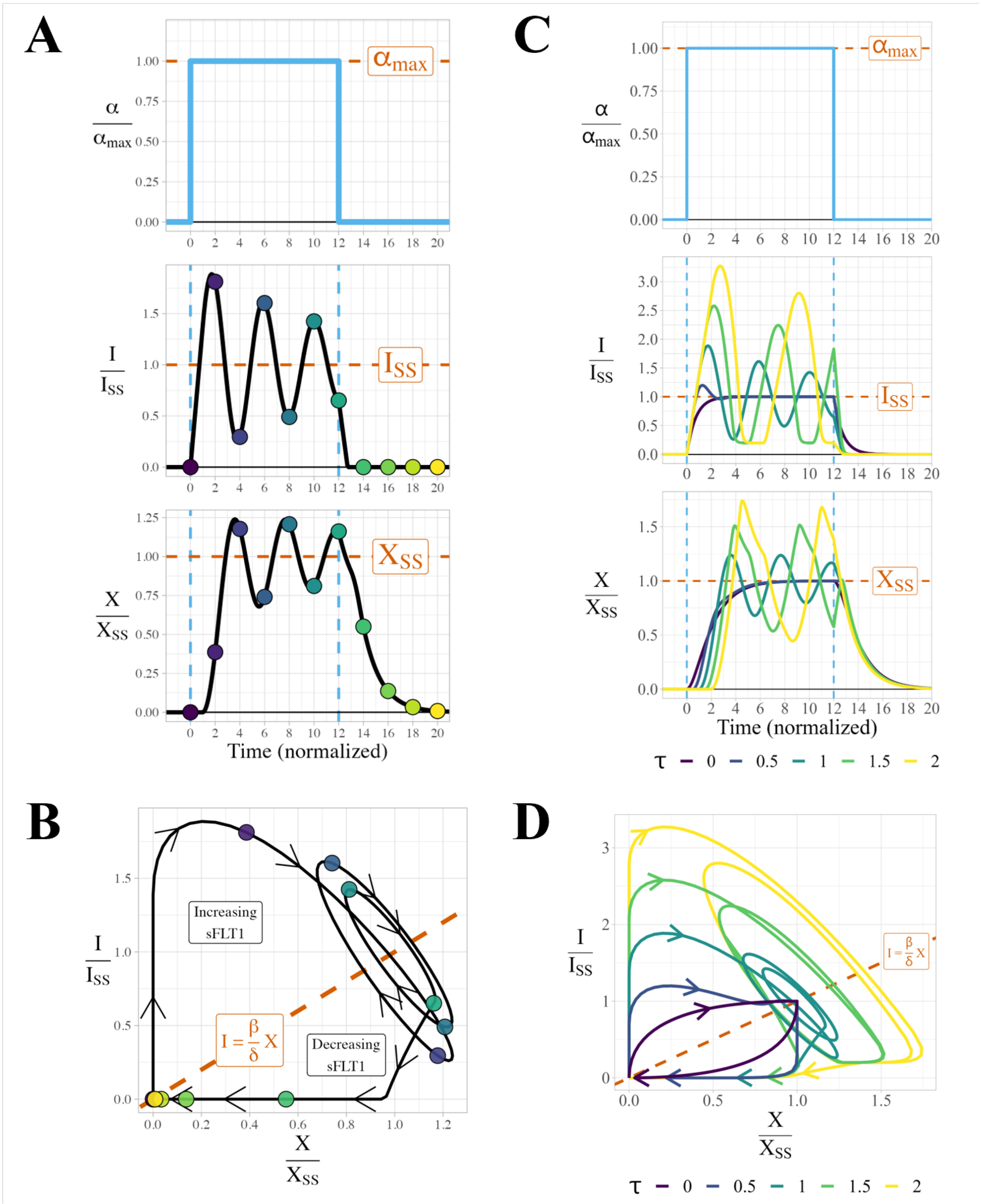

S2 Fig. Dynamics and phase plane behavior of an example DDE model solution. (A) Solution of the DDE model as a function of time showing intracellular (*I*) and extracellular (*X*) sFLT1 protein dynamics in response to step changes in sFLT1 production (α). Time is normalized to T_{50_X}, the half-life of X (Eq. 4). I, X are normalized to their

steady state values I_{SS}, X_{SS} (Eq. 1-2). **(B)** Phase portrait of the shown DDE model solution. Points are evenly spaced every two time units and colored by time as in panel A. Arrowheads indicate the direction of increasing time starting with $t=0$ in the lower left. The dashed identity line represents potential steady states ($I = \frac{\beta}{\delta} \cdot X$); total sFLT1 increases in regions above this line and decreases below. Parameter values: $\alpha = 10{,}000$, $\beta = 0.1$, $\gamma = 0.1$, $\delta = 0.1$, $\tau = T_{50\_X}$. **(C)** Example DDE solutions as a function of time and **(D)** as a phase portrait for maturation delays $\tau = (0, 0.5, 1, 1.5, 2) \cdot T_{50\_X}$. For discussion of this figure, see ***S2 Text**, Supplemental Results*.
