# Supplementary material for: Mechanistic computational modeling of sFLT1 secretion dynamics
Source: PLoS Comput Biol. 2025 Aug 18;21(8):e1013324. doi: 10.1371/journal.pcbi.1013324 (PMC12370208; doi:10.1371/journal.pcbi.1013324)
Supplement: S3 Fig — Comparison of experimental data (points) and simulated time courses (lines, n=100 per plot) of (A, C) extracellular and (B, D) intracellular sFLT1 for (A, B) pulse-chase secretion and (C, D) constitutive secretion cases using eight distinct candidate models as described in Fig 3A and S1 Table. In pulse-chase plots, the highlighted region (P) marks the 20-minute pulse. X/X8h: extracellular sFLT1 normalized to its value at 8h; X/X24h: extracellular sFLT1 normalized to its value at 24h; I/I0h: intracellular sFLT1 normalized to its value at t=0. (PDF) [file pcbi.1013324.s010.pdf]

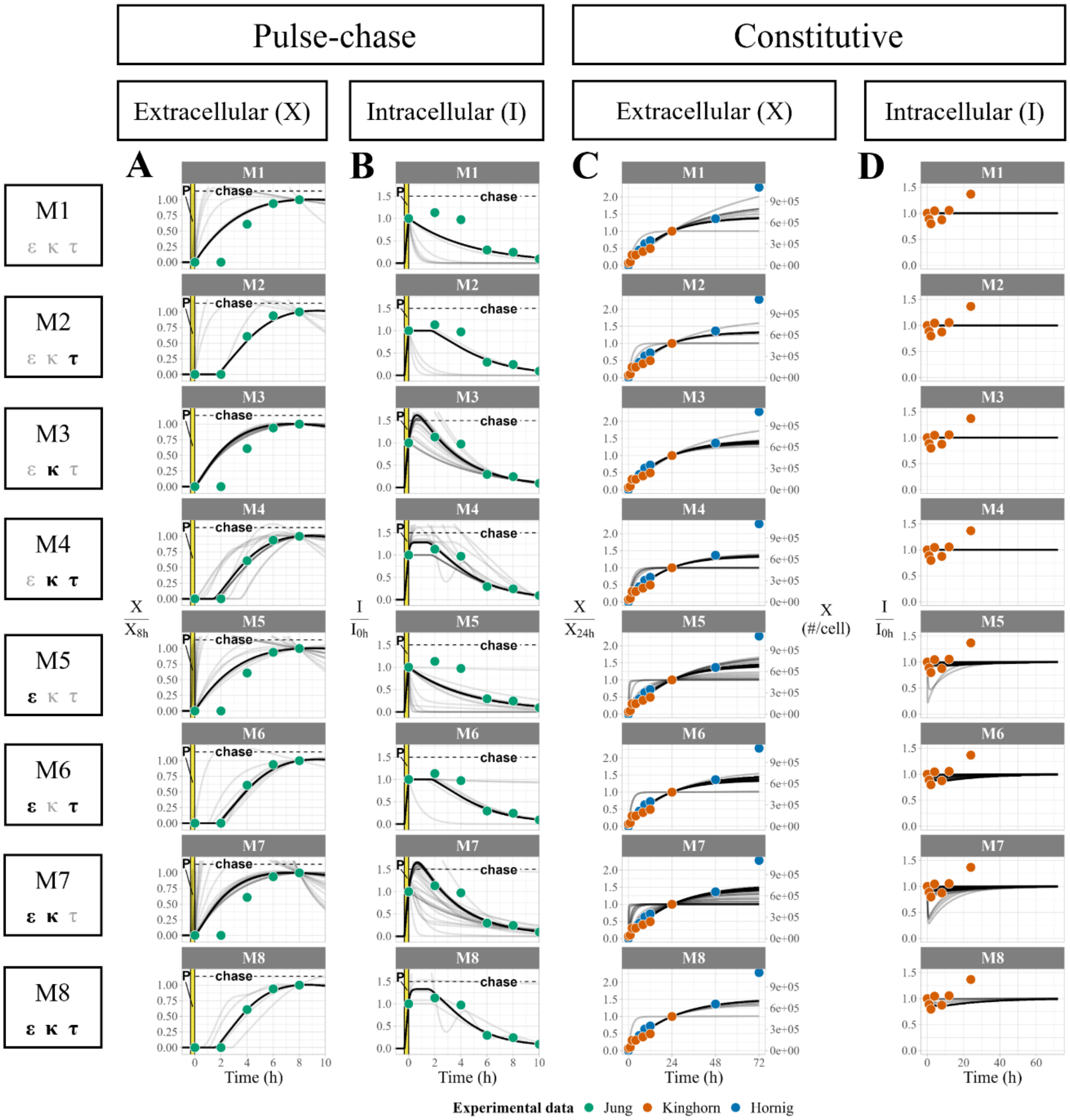

S3 Fig. Visual predictive checks of candidate models of sFLT1 secretion. Comparison of experimental data (points) and simulated time courses (lines, $n = 100$ per plot) of **(A, C)** extracellular and **(B, D)** intracellular sFLT1 for **(A, B)** pulse-chase secretion and **(C, D)** constitutive secretion cases using eight distinct candidate models as described in **Fig 3A** and **S1 Table**. In pulse-chase plots, the highlighted region (P) marks the 20-minute pulse. X/X_{8h}: extracellular sFLT1 normalized to its value at 8h; X/X_{24h}: extracellular sFLT1 normalized to its value at 24h; I/I_{0h}: intracellular sFLT1 normalized to its value at $t = 0$.
