# Supplementary material for: Mechanistic computational modeling of sFLT1 secretion dynamics
Source: PLoS Comput Biol. 2025 Aug 18;21(8):e1013324. doi: 10.1371/journal.pcbi.1013324 (PMC12370208; doi:10.1371/journal.pcbi.1013324)
Supplement: S4 Fig — (A) Visual predictive checks of unfiltered optimization runs (n=1000) from the delay differential equation (DDE) model. Many runs with high cost have not converged to follow the experimental data. (B) Cost distribution of raw (left) and filtered (right) optimization runs of the DDE model. The red lines indicate the cost cutoff of 10% above the minimum cost. (C) Density plots of raw and filtered initial parameter values for the DDE model. Raw (yellow) distributions include all attempted optimizations (n=1000). Filtered (black) distributions include only fits with a cost within 10% of the best fit (n=594). Y axes are normalized such that each distribution has a maximum density of 1. (PDF) [file pcbi.1013324.s011.pdf]

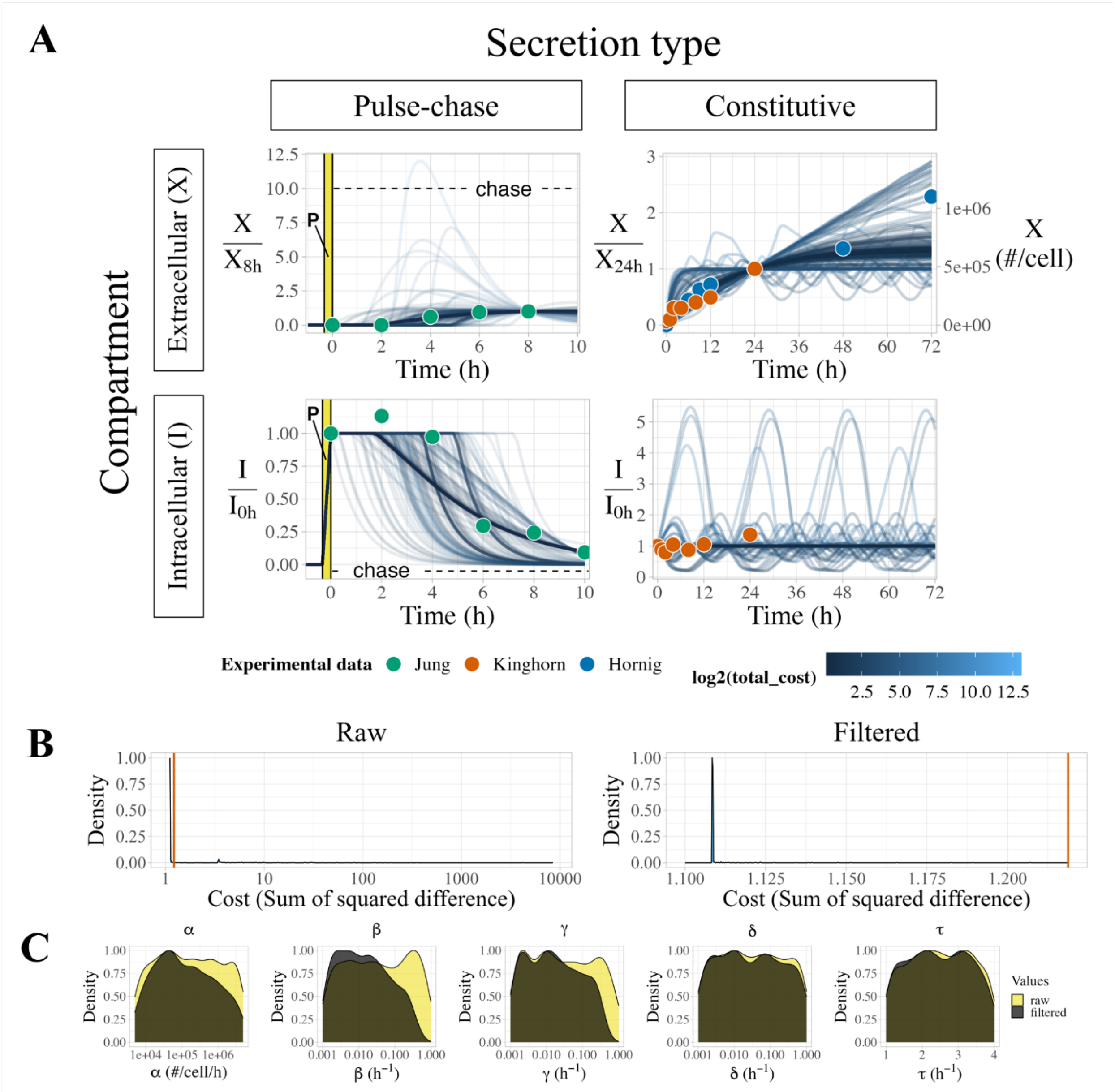

S4 Fig. Filtering DDE optimization runs by cost. (A) Visual predictive checks of unfiltered optimization runs ($n = 1000$) from the delay differential equation (DDE) model. Many runs with high cost have not converged to follow the experimental data. **(B)** Cost distribution of raw (left) and filtered (right) optimization runs of the DDE model. The red lines indicate the cost cutoff of 10% above the minimum cost. **(C)** Density plots of raw and filtered initial parameter values for the DDE model. Raw (yellow) distributions include all attempted optimizations ($n = 1000$). Filtered (black) distributions include only fits with a cost within 10% of the best fit ($n = 594$). Y axes are normalized such that each distribution has a maximum density of 1.
