# Supplementary material for: Mechanistic computational modeling of sFLT1 secretion dynamics
Source: PLoS Comput Biol. 2025 Aug 18;21(8):e1013324. doi: 10.1371/journal.pcbi.1013324 (PMC12370208; doi:10.1371/journal.pcbi.1013324)
Supplement: S5 Fig — (A) Violin plots of optimal parameter values (n=594) for the delay differential equation (DDE) model. Units: α, #/cell/h; (β,γ,δ,ε), h-1. (B) Correlations between δ and τ and other optimized parameters. Each point represents the observed values of the listed parameters in a single run of the delay differential equation model after filtering for low-cost fits (n=594). (PDF) [file pcbi.1013324.s012.pdf]

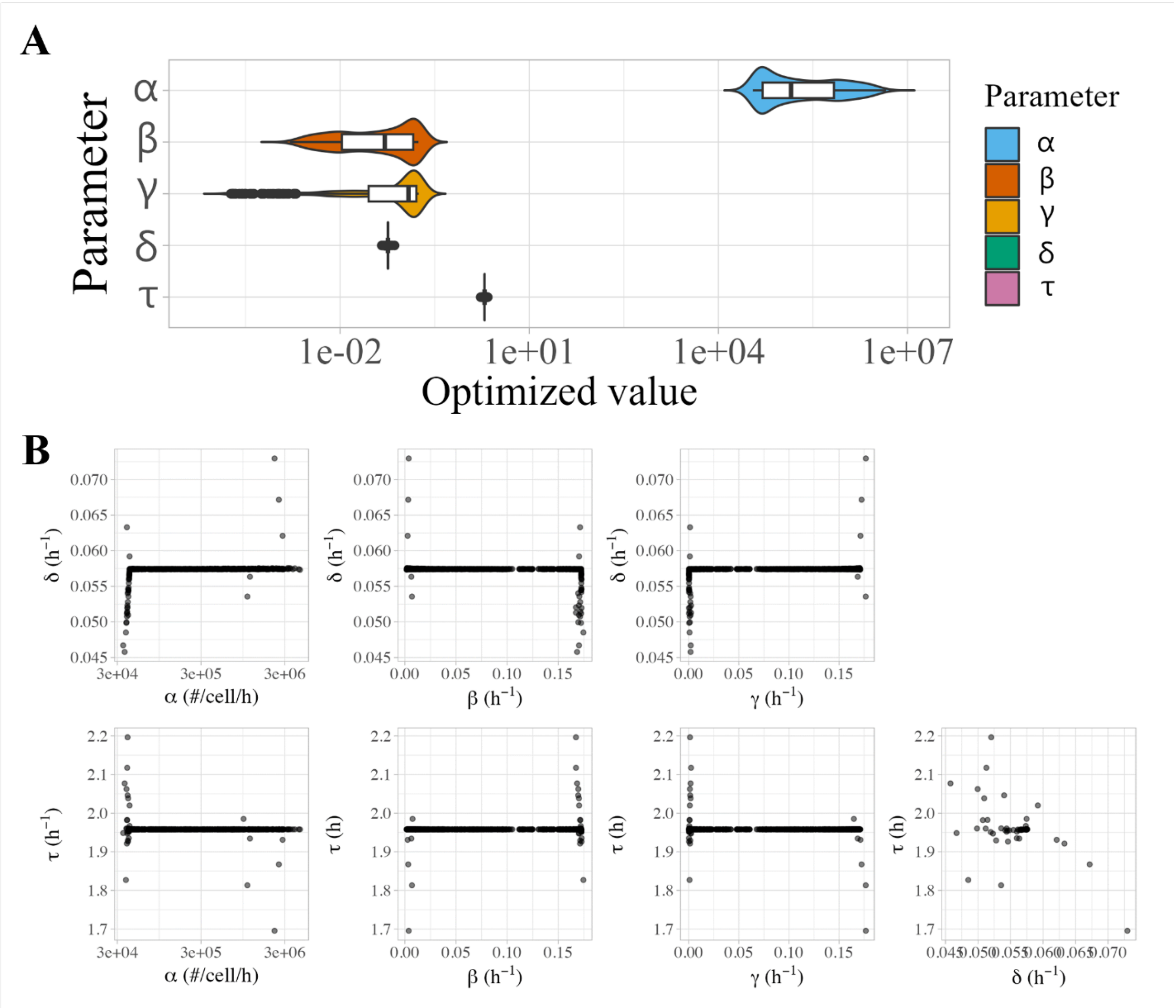

S5 Fig. Properties of optimized parameters from the DDE model of sFLT1 secretion. (A) Violin plots of optimal parameter values ($n = 594$) for the delay differential equation (DDE) model. Units: α, #/cell/h; (β,γ,δ,ε), h^{-1}. **(B)** Correlations between δ and τ and other optimized parameters. Each point represents the observed values of the listed parameters in a single run of the delay differential equation model after filtering for low-cost fits ($n = 594$).
