# Supplementary material for: Mechanistic computational modeling of sFLT1 secretion dynamics
Source: PLoS Comput Biol. 2025 Aug 18;21(8):e1013324. doi: 10.1371/journal.pcbi.1013324 (PMC12370208; doi:10.1371/journal.pcbi.1013324)
Supplement: S6 Fig — (A) Time courses of absolute intracellular sFLT1 (I) during simulation of constitutive secretion. (B) Distribution of steady state values of intracellular sFLT1 (ISS). The dashed red line indicates the theoretical lower bound min(ISS) =c1/c22 based on median values of c1 and c2 (Table 2). (PDF) [file pcbi.1013324.s013.pdf]

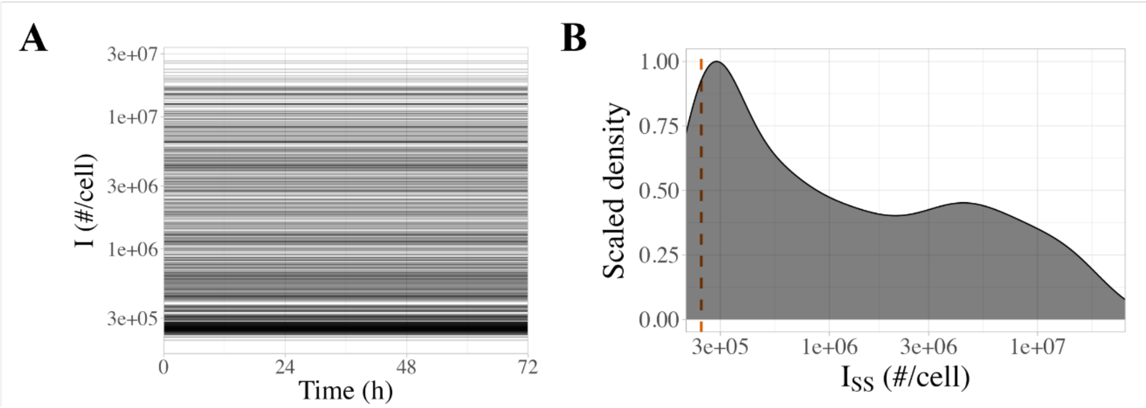

S6 Fig. Simulated absolute numbers of intracellular sFLT1 molecules. (A) Time courses of absolute intracellular sFLT1 (*I*) during simulation of constitutive secretion. **(B)** Distribution of steady state values of intracellular sFLT1 (I_{SS}). The dashed red line indicates the theoretical lower bound $\min(I_{SS}) = c_1/c_2^2$ based on median values of c_1 and c_2 (**Table 2**).
