# Supplementary material for: Mechanistic computational modeling of sFLT1 secretion dynamics
Source: PLoS Comput Biol. 2025 Aug 18;21(8):e1013324. doi: 10.1371/journal.pcbi.1013324 (PMC12370208; doi:10.1371/journal.pcbi.1013324)
Supplement: S7 Fig — All annotation lines are derived in S1 Text and calculated using median values of c1 and c2. (A) Correlation between secretion flux (Φ(Secr)) and the compound parameter αβ/(β+γ)=c1/c2. Both dashed lines indicate c1/c2. (B) Correlation between intracellular degradation flux (Φ(IDeg)) and production flux (Φ(Prod)). Dashed lines indicate the theoretical lower bound min(Prod) =c1/c2 and the asymptote Φ(IDeg) = Φ(Prod). (C) Correlation between steady state intracellular sFLT1 (ISS) and intracellular degradation flux (Φ(IDeg)). Dashed lines indicate the theoretical lower bound min(ISS) =c1/c22 and the asymptote ISS= Φ(IDeg)/c2. (D) Correlation between steady state intracellular sFLT1 (ISS) and production flux (Φ(Prod)) during constitutive secretion. Dashed lines indicate theoretical lower bounds (min(ISS) =c1/c22) and min(Φ(Prod)) =c1/c2). Φ = Flux, Prod = production, Secr = secretion, IDeg = intracellular degradation, XDeg = extracellular degradation. (PDF) [file pcbi.1013324.s014.pdf]

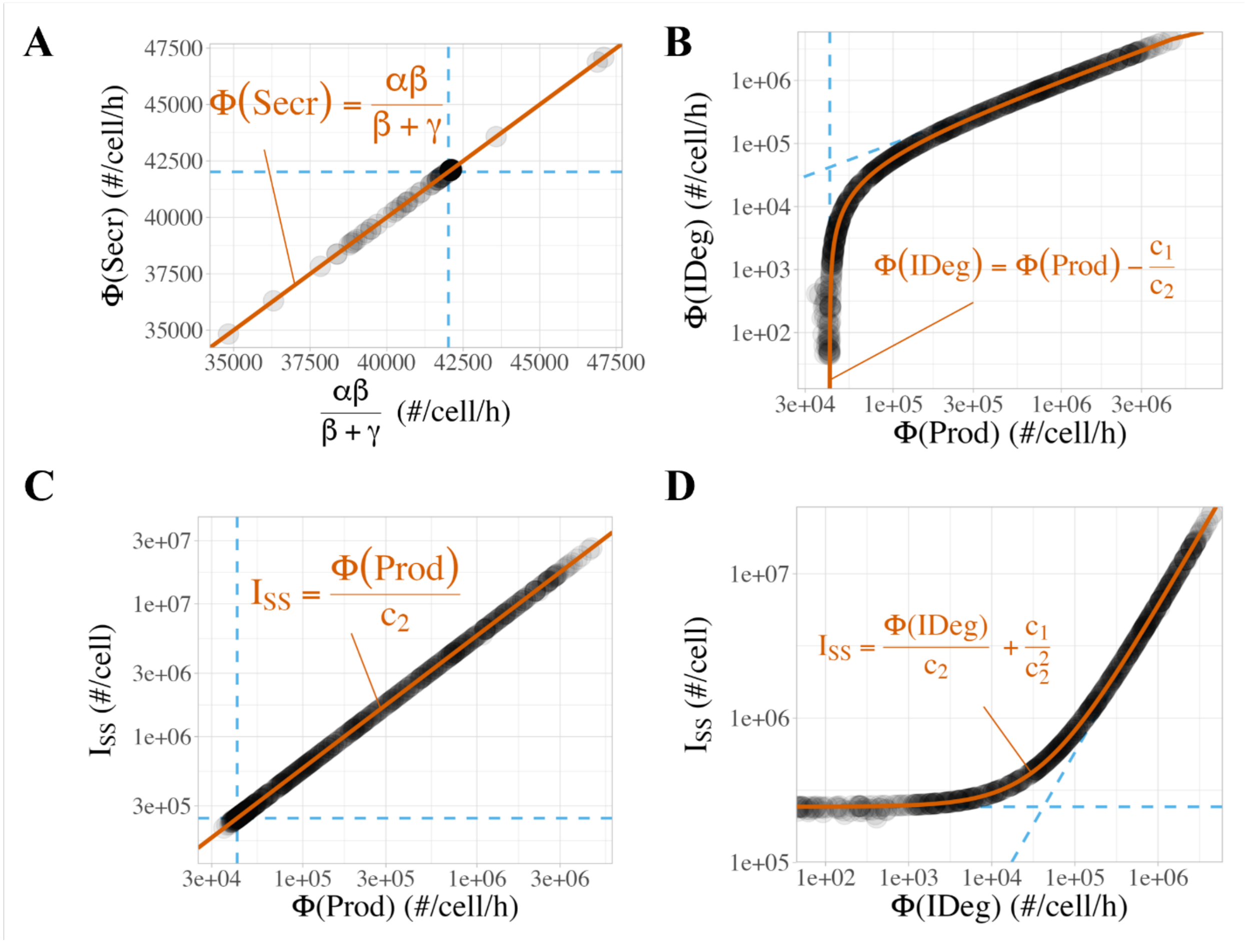

S7 Fig. Steady state properties of process fluxes during simulation of constitutive secretion. All annotation lines are derived in *S1 Text, Supplemental Methods* and calculated using median values of c_1 and c_2. **(A)** Correlation between secretion flux (Φ(Secr)) and the compound parameter $\alpha\beta/(\beta+\gamma) = c_1/c_2$. Both dashed lines indicate c_1/c_2 . **(B)** Correlation between intracellular degradation flux (Φ(IDeg)) and production flux (Φ(Prod)). Dashed lines indicate the theoretical lower bound min(Prod) = c_1/c_2 and the asymptote Φ(IDeg) = Φ(Prod). **(C)** Correlation between steady state intracellular sFLT1 (I_{SS}) and intracellular degradation flux (Φ(IDeg)). Dashed lines indicate the theoretical lower bound $\min(I_{SS}) = c_1/c_2^2$ and the asymptote $I_{SS} = \Phi(\text{IDeg})/c_2$. **(D)** Correlation between steady state intracellular sFLT1 (I_{SS}) and production flux (Φ(Prod)) during constitutive secretion. Dashed lines indicate theoretical lower bounds ($\min(I_{SS}) = c_1/c_2^2$) and $\min(\Phi(\text{Prod})) = c_1/c_2$). Φ = Flux, Prod = production, Secr = secretion, IDeg = intracellular degradation, XDeg = extracellular degradation.
