# Supplementary material for: Mechanistic computational modeling of sFLT1 secretion dynamics
Source: PLoS Comput Biol. 2025 Aug 18;21(8):e1013324. doi: 10.1371/journal.pcbi.1013324 (PMC12370208; doi:10.1371/journal.pcbi.1013324)
Supplement: S8 Fig — All species are normalized to their maximum observed values over 10 hours. Φ = flux, Prod = production, Secr = secretion, IDeg = intracellular degradation, XDeg = extracellular degradation. (PDF) [file pcbi.1013324.s015.pdf]

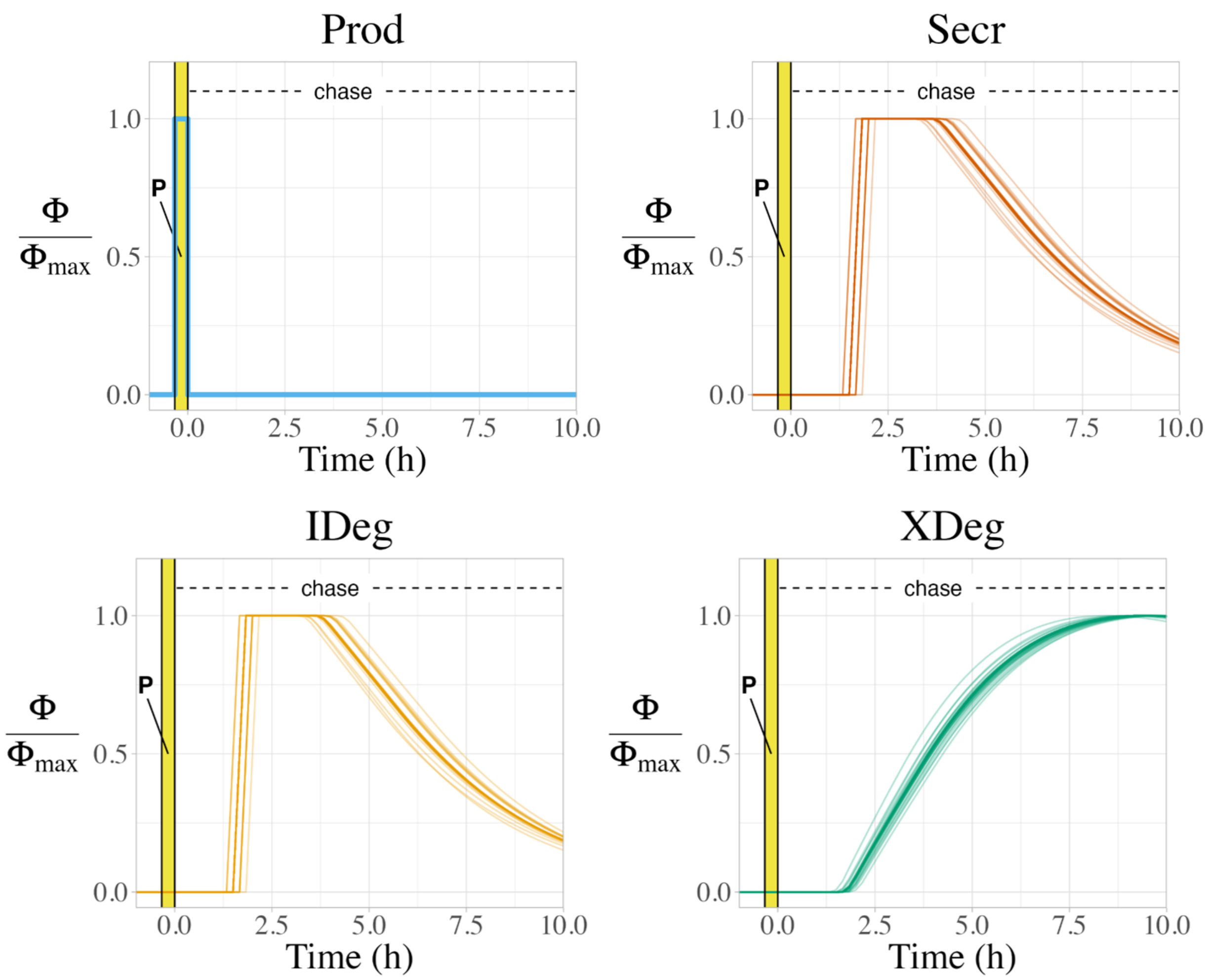

S8 Fig. Relative process fluxes over time during simulations of pulse-chase secretion. All species are normalized to their maximum observed values over 10 hours. Φ = flux, Prod = production, Secr = secretion, IDeg = intracellular degradation, XDeg = extracellular degradation.
