# Supplementary material for: Mechanistic computational modeling of sFLT1 secretion dynamics
Source: PLoS Comput Biol. 2025 Aug 18;21(8):e1013324. doi: 10.1371/journal.pcbi.1013324 (PMC12370208; doi:10.1371/journal.pcbi.1013324)
Supplement: S9 Fig — Top: extracellular sFLT1 at 72 hours (X72h); bottom: steady state intracellular sFLT1 (ISS). (PDF) [file pcbi.1013324.s016.pdf]

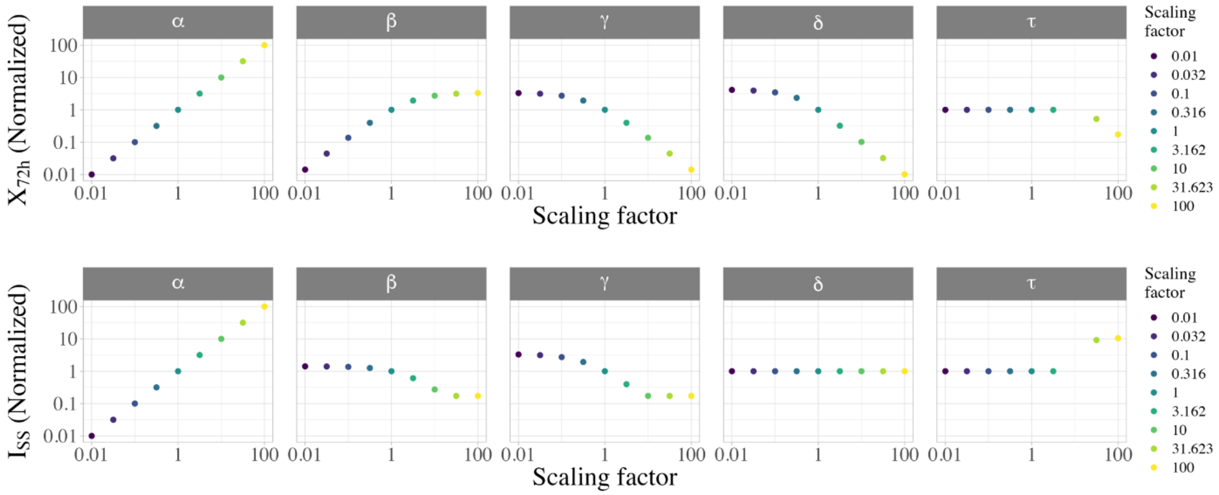

S9 Fig. Impact of individual parameter variations on sFLT1 for simulations of constitutive secretion. Top: extracellular sFLT1 at 72 hours (X_{72h}); bottom: steady state intracellular sFLT1 (I_{SS}).
