# Supplementary material for: Mechanistic computational modeling of sFLT1 secretion dynamics
Source: PLoS Comput Biol. 2025 Aug 18;21(8):e1013324. doi: 10.1371/journal.pcbi.1013324 (PMC12370208; doi:10.1371/journal.pcbi.1013324)
Supplement: S10 Fig — (A) Time courses of extracellular (X) and intracellular (I) sFLT1 during constitutive secretion with fixed c1=7270 #/cell/h [2] and varying c2. (B) Correlation between extracellular sFLT1 at 72 hours (X72h) or steady state intracellular sFLT1 (ISS) with β, γ, and c2 when c1 is constant during constitutive secretion. (C) Time courses of extracellular (X) and intracellular (I) sFLT1 during constitutive secretion with fixed c2=0.173 h-1 and varying c1. (D) Correlation between extracellular sFLT1 at 72 hours (X72h) with c1, β, and γ when c2 is constant during constitutive secretion. (PDF) [file pcbi.1013324.s017.pdf]

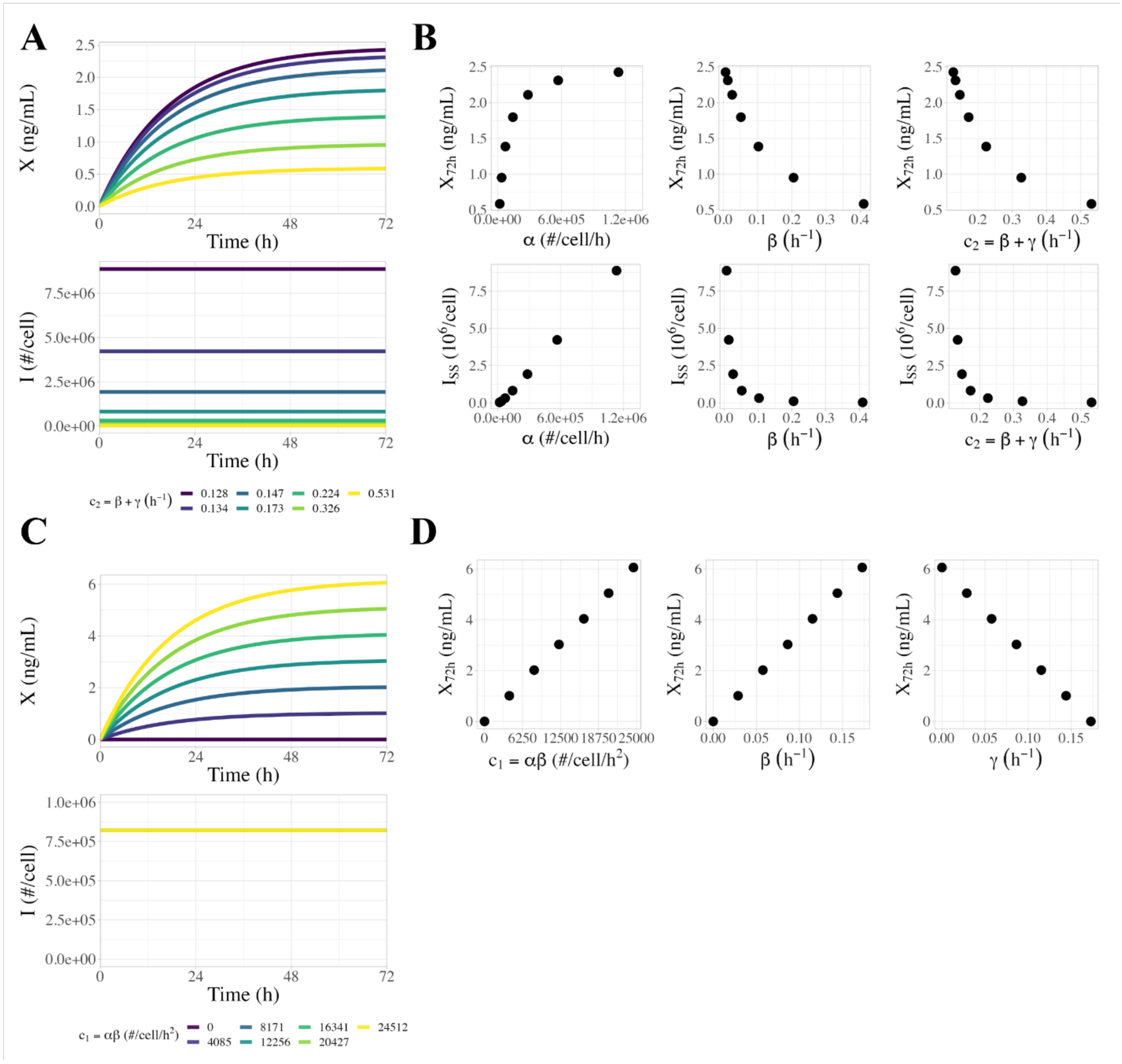


**S10 Fig. Sensitivity of extracellular and intracellular sFLT1 to the compound parameters $c_1 = \alpha\beta$ and $c_2 = \beta + \gamma$ in the DDE model. (A)** Time courses of extracellular ($X$) and intracellular ($I$) sFLT1 during constitutive secretion with fixed $c_1 = 7270$ #/cell/h$^2$ and varying $c_2$. **(B)** Correlation between extracellular sFLT1 at 72 hours ($X_{72h}$) or steady state intracellular sFLT1 ($I_{SS}$) with $\alpha$, $\beta$, and $c_2$ when $c_1$ is constant during constitutive secretion. **(C)** Time courses of extracellular (X) and intracellular (I) sFLT1 during constitutive secretion with fixed $c_2 = 0.173$ h$^{-1}$ and varying $c_1$. **(D)** Correlation between extracellular sFLT1 at 72 hours ($X_{72h}$) with $c_1$, $\beta$, and $\gamma$ when $c_2$ is constant during constitutive secretion.
