# Supplementary material for: Mechanistic computational modeling of sFLT1 secretion dynamics
Source: PLoS Comput Biol. 2025 Aug 18;21(8):e1013324. doi: 10.1371/journal.pcbi.1013324 (PMC12370208; doi:10.1371/journal.pcbi.1013324)
Supplement: S11 Fig — Change in extracellular (X) and intracellular (I) sFLT1 at 18 hours (solid) or 72 hours (dashed) with (A) chemical inhibition or (B) genetic downregulation of individual parameters at varying fraction inhibition. sFLT1 values are normalized to the 18-hour (solid) or 72-hour (dashed) values from simulation with the median parameter set. (PDF) [file pcbi.1013324.s018.pdf]

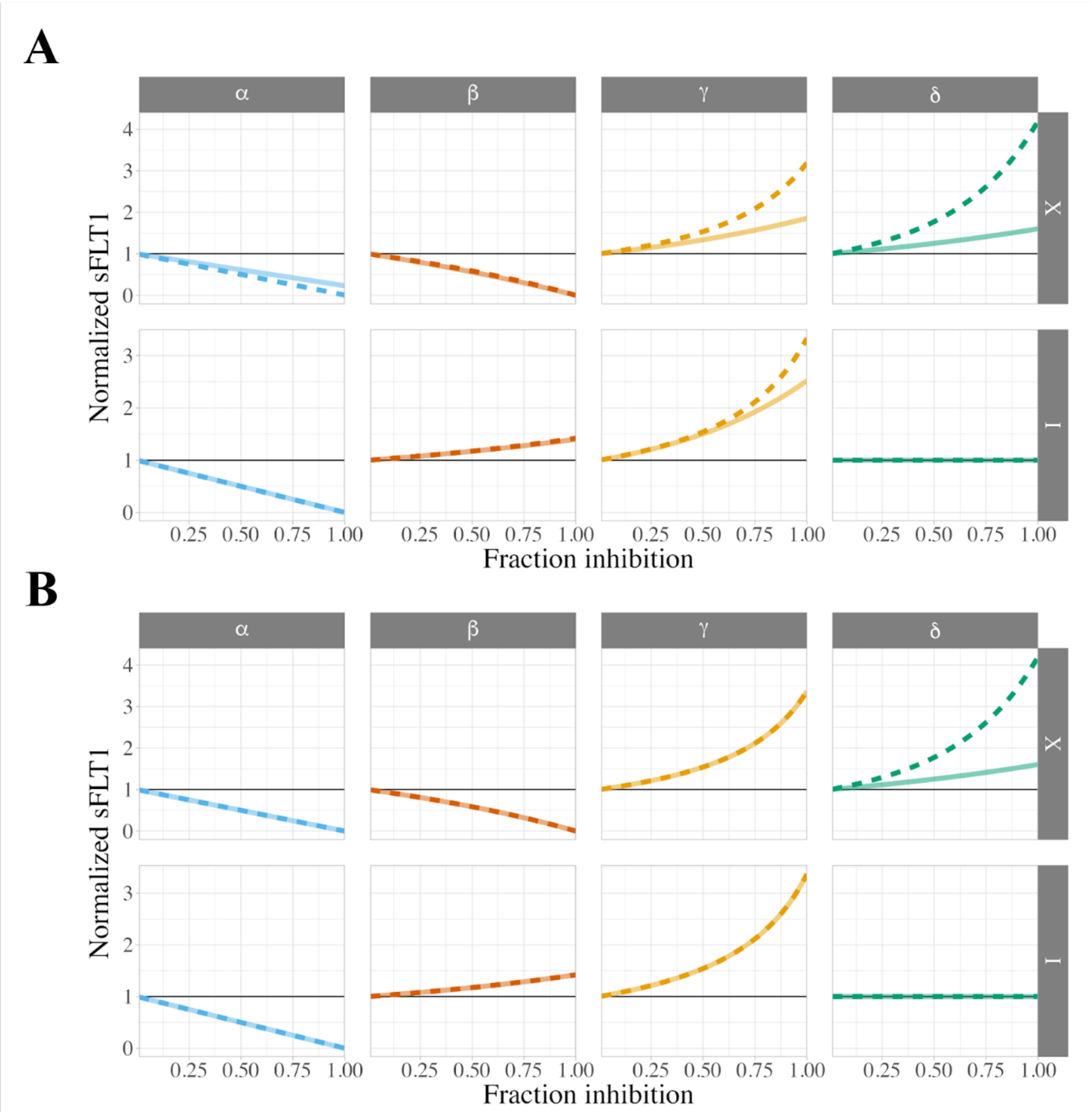

S11 Fig. Simulating different strengths of chemical inhibition and genetic downregulation of sFLT1 secretion. Change in extracellular (X) and intracellular (I) sFLT1 at 18 hours (solid) or 72 hours (dashed) with **(A)** chemical inhibition or **(B)** genetic downregulation of individual parameters at varying fraction inhibition. sFLT1 values are normalized to the 18-hour (solid) or 72-hour (dashed) values from simulation with the median parameter set.
