# Supplementary material for: Mechanistic computational modeling of sFLT1 secretion dynamics
Source: PLoS Comput Biol. 2025 Aug 18;21(8):e1013324. doi: 10.1371/journal.pcbi.1013324 (PMC12370208; doi:10.1371/journal.pcbi.1013324)
Supplement: S12 Fig — Time courses of extracellular (X) and intracellular (I) sFLT1 during constitutive simulation with different base parameter sets sharing constraints of c1=αβ=7270 #/cell/h [2] and c2=β+γ=0.173 h-1. These time courses are used as the base cases for testing chemical and genetic inhibition from different initial conditions (Figs 9 and S13). (PDF) [file pcbi.1013324.s019.pdf]

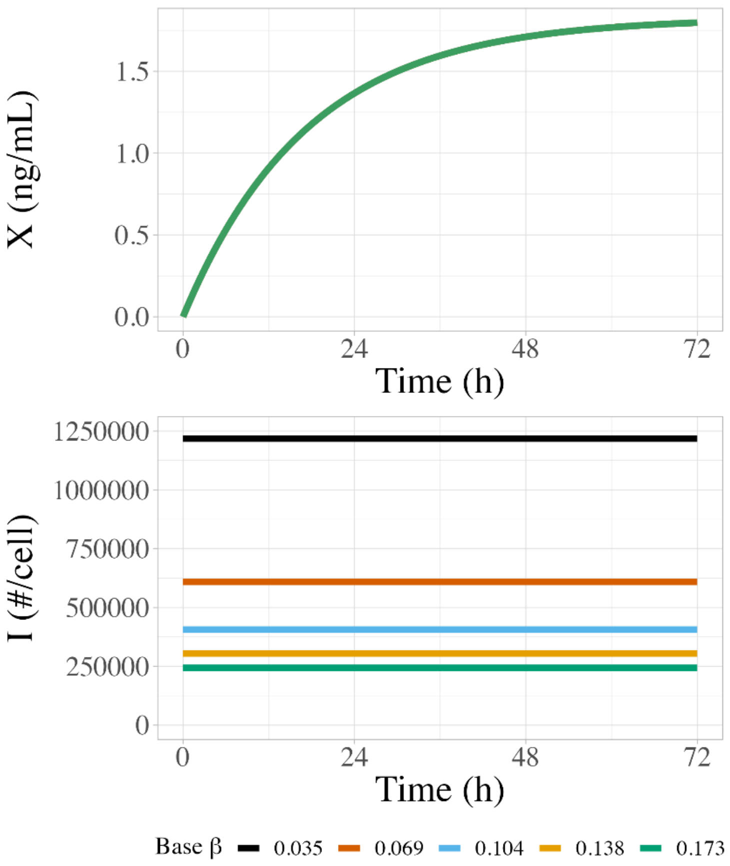

S12 Fig. Different sets of initial conditions for simulating chemical and genetic inhibition. Time courses of extracellular (*X*) and intracellular (*I*) sFLT1 during constitutive simulation with different base parameter sets sharing constraints of $c_1 = \alpha\beta = 7270$ #/cell/h^2 and $c_2 = \beta + \gamma = 0.173$ h^{-1}. These time courses are used as the base cases for testing chemical and genetic inhibition from different initial conditions (**Figs 9 and S13**).
