# Supplementary material for: Mechanistic computational modeling of sFLT1 secretion dynamics
Source: PLoS Comput Biol. 2025 Aug 18;21(8):e1013324. doi: 10.1371/journal.pcbi.1013324 (PMC12370208; doi:10.1371/journal.pcbi.1013324)
Supplement: S13 Fig — (A) Change in extracellular (X) and intracellular (I) sFLT1 at 18 hours (top) and 72 hours (bottom) with chemical inhibition of individual parameters at varying fraction inhibition for different base parameter sets (labeled by β values). (B) Change in extracellular (X) and intracellular (I) sFLT1 at 18 hours (top) and 72 hours (bottom) post media change with genetic inhibition of individual parameters at varying fraction inhibition for different base parameter sets (labeled by β values). X and I are normalized to the values at the end of each time course (18h or 72h, respectively) from the simulation with the corresponding base parameter set. (PDF) [file pcbi.1013324.s020.pdf]

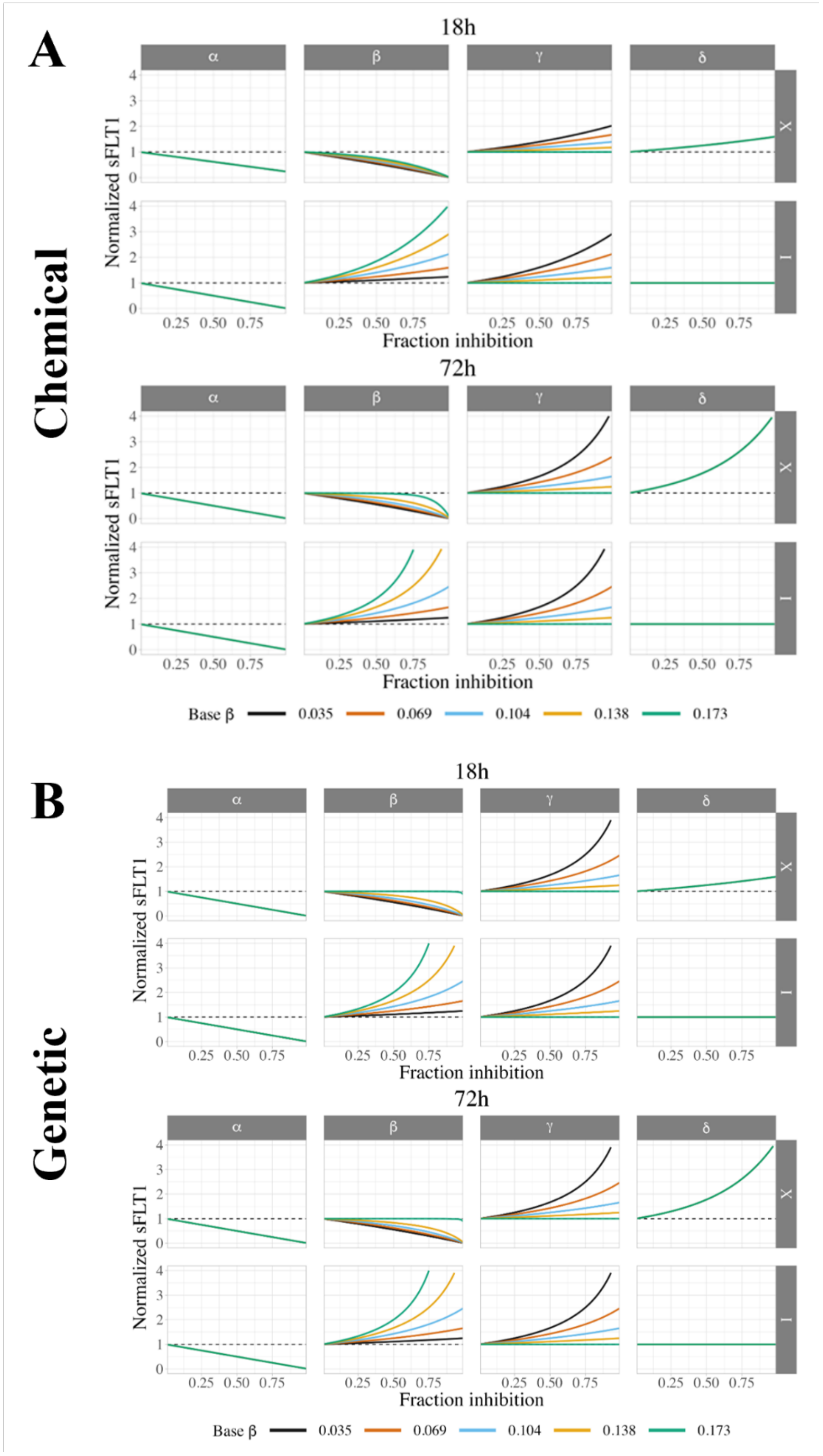

S13 Fig. Variation in predicted effects of chemical and genetic perturbations across optimized parameter sets. **(A)** Change in extracellular (*X*) and intracellular (*I*) sFLT1 at 18 hours (top) and 72 hours (bottom) with chemical inhibition of individual parameters at varying fraction inhibition for different base parameter sets (labeled by β values). **(B)** Change in extracellular (*X*) and intracellular (*I*) sFLT1 at 18 hours (top) and 72 hours (bottom) post

media change with genetic inhibition of individual parameters at varying fraction inhibition for different base parameter sets (labeled by β values). X and I are normalized to the values at the end of each time course (18h or 72h, respectively) from the simulation with the corresponding base parameter set.
