# Supplementary material for: Mechanistic computational modeling of sFLT1 secretion dynamics
Source: PLoS Comput Biol. 2025 Aug 18;21(8):e1013324. doi: 10.1371/journal.pcbi.1013324 (PMC12370208; doi:10.1371/journal.pcbi.1013324)
Supplement: Attachment — Submitted filename: Response to Reviewers.pdf [file pcbi.1013324.s022.pdf]

Amy Gill
Institute for Computational Medicine
Department of Biomedical Engineering
Johns Hopkins University
agill18@jhmi.edu

June 12, 2025

Dear Editor,

We enclose for your consideration our response to the reviews of our manuscript. We thank the editors and reviewers for the constructive feedback they provided. We have carefully addressed the reviewers' critiques as well as the journal requirements. Below we provide a point-by-point response to the points raised by the reviewers. We hope that you and the reviewers will find our manuscript to be improved and now suitable for publication in *PLOS Computational Biology*.

With thanks,
Amy Gill on behalf of the co-authors

REVIEWER #1

"The authors describe 8 mechanistic models for the production and secretion of sFLT1 involved in VEGF signaling. Comparing these models to existing experimental sFLT1 data, the authors identify the appropriate models and parameters. The manuscript is clearly written and describes the modeling process, choice of models, parameter variations, the choice of the model best fitting the data, study conclusions and limitations. I especially praise the full documentation of the models available on GitHub."

We thank the reviewer for their comments.

"The use of figures is non-sequential, with Figure 1A first, then Figure 2A, then Figure 1B, etc."

While we agree that this is somewhat unusual, it is a result of a few references to figure schematic panels (1A, 2A) in the Methods section. Within the Results section, the panels are referenced in sequential order (Figure 1A, 1B, 2A, 2B, 3A, etc.), and overall in the paper, the figures are cited in numerical order, which is the journal requirement. After careful consideration, we decided to leave the minimal figure references in the Methods in the original order, as we believe this helps link the methods to the appropriate figure panels; and we left the schematic panels as 1A, 2A etc. because it fits best for the figure arrangements.

“Figure 3A should also be cited on page 10, when the authors start talking about candidate models.”

We have added this figure citation (p. 10).

“What are ‘model A’ and ‘model B’ in Supplemental Table S1? Should be M1 and M2.”

The reviewer is correct; we have now corrected this error in the caption for Supplemental Table S1.

For data extracted from published figures: “…provide the data in the tabular form. Please be more specific about which figure and which panel [were] used for data extraction. Please refer to specific tables/figures in Jung, Hornig, and Kinghorn studies, and repeat this data in supplementary material, with captions stating what was omitted.”

We now provide the extracted data in Supplementary Data S1 (cited p. 13), an Excel spreadsheet which contains data from all three studies with explicit reference to the figure panel of the original publication, as well as noting omitted data points and the rationale for omission. Notes on data point omissions remain included in Supplemental Table S2.

“What is ‘custom cost function’ on page 13?”

We have edited this phrase to “cost function described above” (p. 15), as the cost function is introduced in the previous subsection titled “Optimization cost function” (pp. 14-15). That cost function section has also been rewritten in response to a comment from Reviewer 2, as described below.

“Please remind the user of the values of n and k for each M1-M8 models.”

We have updated the caption of Figure 3B:

> “All models are fit to the same number of observations (n=26), while the number of parameters *k* varies by model.”

We have also updated the x-axis of Figure 3C to clarify that the number of parameters shown in this plot is the k value for the AIC calculation.

“Figure 3B – would be useful to name columns M1-M8.”

We have added a second set of column names to the top of Figure 3B to complement those at the bottom of the panel.

"Page 24: 'we chemically inhibited each parameter individually…' – perhaps the author meant to say they simulated inhibition of each individual parameter by 90%."

We have altered this to: "we simulated chemical inhibition of each individual parameter…" (p. 28).

"Page 25: 'lysosomal degradation inhibitor chloroquine' and 'the Golgi inhibitor brefeldin' – it's the first time these inhibitors are introduced, authors need to put them into the context of models M1-M8."

We have added the following information (p. 30) to clarify how these inhibitors relate to our model rate constants:

> "Four chemical inhibitors were considered: brefeldin, a Golgi inhibitor that blocks protein maturation and secretion, described in our model as a component of the secretion rate constant β (a rate constant that combines all steps of secretion); chlorpromazine, an inhibitor of vesicular transport, described in our model as another component of β; TATNSF700, an inhibitor of exocytosis described in our model as a component of β; and chloroquine, an inhibitor of lysosomal degradation, described in our model as the intracellular degradation rate constant γ."

REVIEWER #2

"This paper describes the development and analysis of a computational model of the secretion dynamics of a soluble form of the VEGF receptor called sFLT1. Data from 5 different experimental studies of the process covering two different experimental scenarios, constitutive and pulse-chase, are integrated into a single calibration set that is used to calibrate and evaluate a family of computational models that contain a set of six common processes and encompassing zero to three additional mechanisms, with the goal of identifying a minimal model that best captures the data. This goal is accomplished by comparing each best fit model using the corrected form of Akaike's Information Criterion (AIC_c), which chooses a model that includes only one of the three proposed additional mechanisms, maturation delay, which is modeled with the addition of a single time delay parameter using delayed differential equations. The paper presents a number of additional analyses of the selected model, including parameter identifiability and sensitivity analysis, which is extended to explore possible mechanisms for chemical and genetic perturbation of the system. A key result of this analysis is that while some

of these effects are well-predicted by the model, others, notably those that are sensitive to intracellular trafficking dynamics, are not. These results provide a strong rationale for future development of models, presumably based on an expanded set of experimental measurements, that more accurately capture these dynamics. Overall, the paper is well-written and clear, and I have just minor comments that I think may improve the manuscript if they are addressed prior to publication. I congratulate the authors on their thorough and rigorous treatment of this system."

We thank the reviewer for their comments.

A general comment is that there are several instances of statements where factual statements are made without a corresponding reference. Examples include:

- p. 27: "...the targets of inhibition can affect the trafficking of many proteins, which may themselves have a role in sFLT1 trafficking."
- p. 34: " Importantly, the chemical and genetic inhibitors explored are not specific to sFLT1, but regulate trafficking for many other proteins, some of which may themselves modulate sFLT1 synthesis and secretion."
- p. 36: " Even in monolayer culture of single cell lines, cells are not identical, as the stochasticity of biochemical reactions leads to natural gradients and fluctuations."

We added citations to these examples, including the following minor edits:

- "For example, STX6 also regulates trafficking of VEGFR2 to and from the cell surface,[37] and VEGF-VEGFR2 signaling induces sFLT1 expression in endothelial cells.[38] Inactivating STX6 thus influences sFLT1 expression through both direct and indirect paths." (p. 34)
- p. 41: Added citations for references 37, 38 without other changes.

- p. 44: "Even in mammalian cell culture of genetically identical cell lines, cell-intrinsic and cell-extrinsic stochastic processes lead to phenotypic differences across the cell population.[42,43]"

We also read the paper to find other clear instances of factual statements without references. We added references for brefeldin inducing the unfolded protein response (p. 41, ref. 40) and the abilities and limitations of agent-based models (p.44, ref. 45).

p. 13. There is a typo in Eq. 5: log is repeated in the first term of the equation. Also, it is more standard to provide the AICc formula as an equation, in other words to write: "AIC_c = n \log (C/n) + 2k + ..." instead of just giving the formula. (Note also that C should be capitalized in the formula.)

The reviewer is correct, and we have corrected Eq. 5 (p. 15): $AIC_C = n\ log\ \left(\frac{C}{n}\right) + 2k + \frac{2k(k+1)}{n-k-1}$

p. 13. " is the cost of a given model calculated using the custom cost function." I think it would be helpful to have an explicit formula for C, referred to in Eq. 5., especially because of the use of the word "custom" here, the reader may think they have missed something.

We have replaced the paragraph describing the cost function as follows (pp. 14-15):

> **Optimization cost function.** For each dataset, we first converted the predicted *X* and *I* values (from the simulations) from units of #/cell to the units measured by the experimental assay. We then compared each observed data point (t_i, y_i) to the corresponding simulated data point $(t_i, f(t_i, p))$ given the adjustable parameter values p. We calculated the cost C as the sum of squared relative errors between observed and simulated values: $C = \sum_{i=1}^{n}\left(v_i - f(t_i, p)\right)^2$. All data points were equally weighted. To find the parameter set p which minimized the cost function, we performed nonlinear least squares optimization using lsqnonlin in MATLAB.

p. 13. In describing use of AIC_c it should be mentioned that there is an additional assumption here that the variance is constant over all the experimental data points and that the additional model parameter is the value of the variance.

We have added the following sentence to end the paragraph (p. 15):

> "This correction factor represents the value of the variance, which is assumed constant over all experimental data points."

p. 19. In Figure 3C labels M4 and M7 are switched.

Nice catch – we missed that! We have corrected the switched labels for M4 and M7 in Figure 3C.

p. 24. "...while extracellular sFLT1 initially increases before declining to a new steady state within 48h." This trend cannot be seen on the graph - maybe show as an inset or in a separate graph.

Panel 7C now includes an inset magnifying the results for chemical inhibition of α. Figure 7F (the corresponding panel for genetic inhibition) also includes an inset at the same scale for

comparison.

p. 30ff. In the Discussion section it would be helpful to refer to specific figures when reviewing the key results of the paper that are being discussed to increase specificity of the points being made. For example, "A key first step in our study was comparing different mechanistic models for sFLT1 secretion [(Fig 2A and 3A)]." "As a result, model M2 - a DDE-based model that included maturation delay but not internalization or production decay - was identified as the best model and was used for the remainder of our study [(Fig 3B,C)]." etc.

The Discussion now contains pointers to the specific figures being discussed.

Fig. 3C - in the combined plot of Cost and AIC there is overlap of the y-axis tick labels for the top and bottom panels. It would help to remove one of the values

We added space between the Cost and AIC barplots and removed the “0” tick from the Cost plot.

Fig. 5D, caption. dX/dt = f(prod)-f(secr) - f(ideg), should be dI/dt.

The reviewer is correct - we have corrected this typo.

Fig. 5E, caption, duplicates 5D caption - shows component fluxes not total.

The reviewer is correct – and the 5F caption was wrong as well. We have corrected the captions for 5E and 5F as follows:

> “(E) Constitutive flux distributions for time-constant processes.”

> “(F) Process fluxes over time during simulation of pulse-chase sFLT1 secretion.”
